# Prostaglandin E_2_ secreted from feline adipose tissue-derived mesenchymal stem cells alleviate DSS-induced colitis by increasing regulatory T cells in mice

**DOI:** 10.1186/s12917-018-1684-9

**Published:** 2018-11-20

**Authors:** Ju-Hyun AN, Woo-Jin SONG, Qiang LI, Sang-Min KIM, Ji-In YANG, Min-Ok RYU, A Ryung NAM, Dong Ha BHANG, Yun-Chan JUNG, Hwa-Young YOUN

**Affiliations:** 1 Labolatory of Veterinary Internal Medicine, Department of Veterinary Clinical Science, College of Veterinary Medicine, Seoul National University, 1 Gwanak-ro, Gwanak-gu, Seoul, 08826 Republic of Korea; 2 Department of Molecular Cell Biology, Samsung Biomedical Research Institute, Sungkyunkwan University School of Medicine, Suwon-si, Gyeonggi-do 16419 Republic of Korea; 3 Chaon Corporation, 335 Pangyo-ro, Bundang-gu, Seongnam-si, Gyeonggi-do 13493 Republic of Korea

**Keywords:** Cytokines, Feline mesenchymal stem cells, Immunomodulation, PBMC, Prostaglandin E_2_, FOXP+ Treg, Inflammatory bowel disease, Colitis

## Abstract

**Background:**

Inflammatory bowel disease (IBD) is an intractable autoimmune disease, relatively common in cats, with chronic vomiting and diarrhea. Previous studies have reported that mesenchymal stem cells (MSCs) alleviate inflammation by modulating immune cells. However, there is a lack of research on cross-talk mechanism between feline adipose tissue-derived mesenchymal stem cells (fAT-MSCs) and immune cells in IBD model. Hence, this study aimed to evaluate the therapeutic effects of fAT-MSC on mice model of colitis and to clarify the therapeutic mechanism of fAT-MSCs.

**Results:**

Intraperitoneal infusion of fAT-MSC ameliorated the clinical and histopathologic severity of colitis, including body weight loss, diarrhea, and inflammation in the colon of Dextran sulfate sodium (DSS)-treated mice (C57BL/6). Since regulatory T cells (Tregs) are pivotal in modulating immune responses and maintaining tolerance in colitis, the relation of Tregs with fAT-MSC-secreted factor was investigated in vitro. PGE_2_ secreted from fAT-MSC was demonstrated to induce elevation of *FOXP3* mRNA expression and adjust inflammatory cytokines in Con A-induced feline peripheral blood mononuclear cells (PBMCs). Furthermore, in vivo, FOXP3+ cells of the fAT-MSC group were significantly increased in the inflamed colon, relative to that in the PBS group.

**Conclusion:**

Our results suggest that PGE_2_ secreted from fAT-MSC can reduce inflammation by increasing FOXP3+ Tregs in mice model of colitis. Consequently, these results propose the possibility of administration of fAT-MSC to cats with not only IBD but also other immune-mediated inflammatory diseases.

**Electronic supplementary material:**

The online version of this article (10.1186/s12917-018-1684-9) contains supplementary material, which is available to authorized users.

## Background

Inflammatory bowel disease (IBD) is the most common intestinal disorder in cats and has been shown to lead to vomiting, chronic diarrhea, and weight loss [1]. Although the exact underlying mechanism remains unknown, possible contributory factors include genetic factors, infectious agents (including bacteria and parasites), allergies (dietary), and immune dysregulation [2, 3]. Treatment of IBD usually involves alteration of the diet and the use of medication, such as immunosuppressants and antibiotics [4]. However, some feline patients do not respond to any of these treatments.

With recent advances in veterinary medicine, stem cell-based treatments have begun to be applied for the treatment of animal inflammatory and immune disorders. Accumulating evidence suggests that the therapeutic potential of mesenchymal stem cells (MSCs) may be attributed to their differentiation and integration into the injured site [5]. Additionally, MSCs have the ability to secrete soluble factors, which functionally modulate the microenvironment of the host tissue to facilitate the endogenous process of immunomodulation [6–8]. Several soluble factors secreted by MSCs, including transforming growth factor-β (TGF-β), indoleamine-pyrrole 2,3-dioxygenase (IDO), nitric oxide (NO), and prostaglandin E_2_ (PGE_2_), have been proposed to mediate the immunosuppressive effect [9, 10]. Previous studies have demonstrated a prominent role of PGE_2_ in the immunomodulatory properties of MSCs [11, 12]; they have proven that PGE_2_ may induce anti-inflammatory activity of MSCs through modulation of regulatory T cells.

Although many studies in veterinary medicine have suggested that MSCs have immunomodulatory effects on activated immune cells [13–20], only a few studies in feline medicine have characterized the secretory factors from feline MSCs. Moreover, the crosstalk mechanisms between feline MSCs and immune cells have not been fully elucidated.

In this study, we examined whether feline adipose tissue-derived mesenchymal stem cells (fAT-MSCs) could alleviate inflammation in colitis in immunocompetent mice induced by dextran sulfate sodium (DSS). Additionally, we analyzed the immunomodulatory mechanisms of PGE_2_ secreted from fAT-MSCs. Our findings provide important insights into the immunomodulatory abilities of the soluble factors of fAT-MSCs.

## Results

### Characterization of fAT-MSCs

**Fig. 1:**
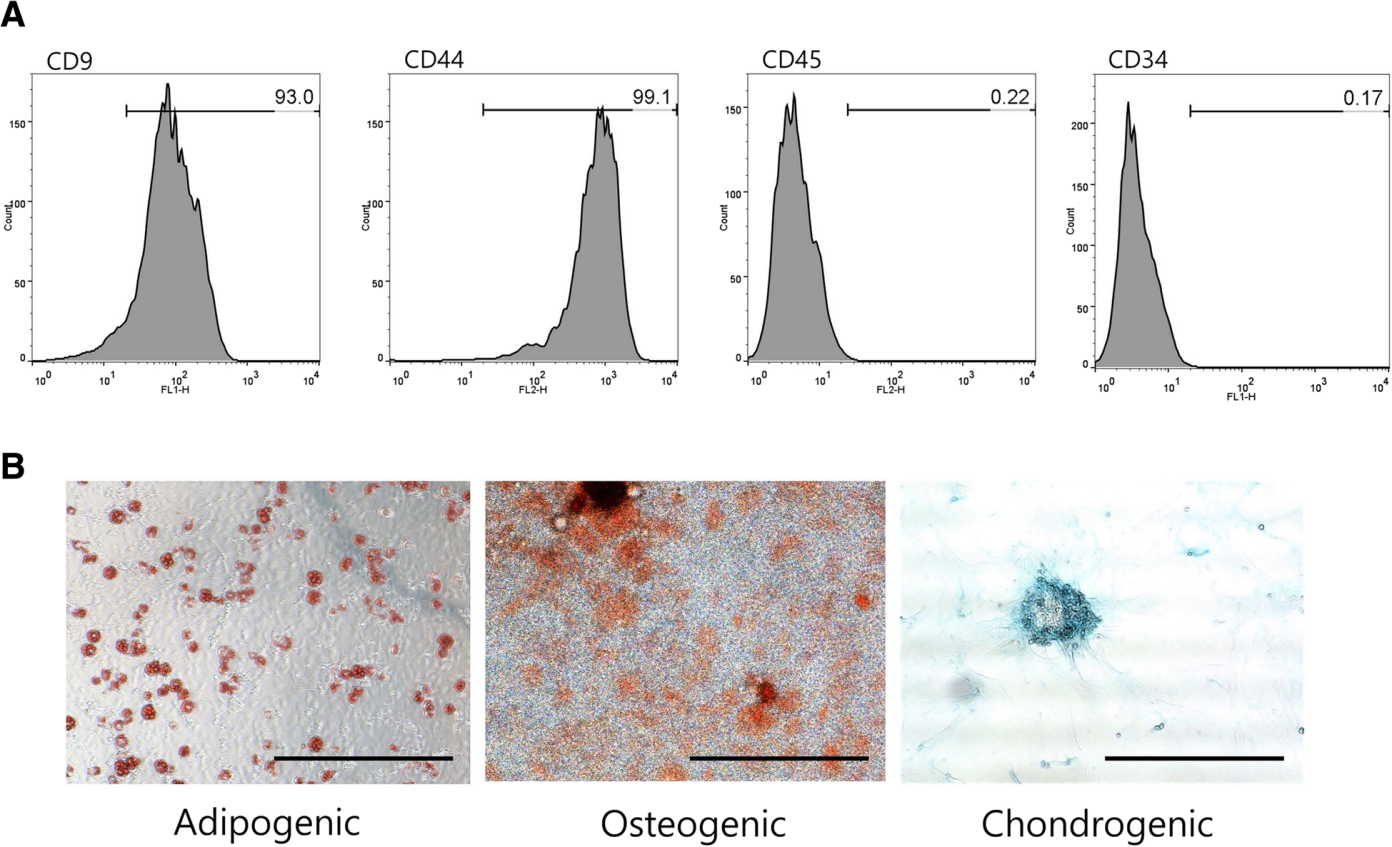
Identification of mesenchymal stem cells (MSCs) isolated from feline adipose tissue. **a** Immunophenotypic analysis by flow cytometry. **b** Adipogenic differentiation; Intracellular lipid vacuoles were stained pink with Oil Red O. Osteogenic differentiation; fAT-MSCs stained positive for calcium deposits with 1% Alizarin red. Chondrogenic differentiation; Proteoglycans were stained with Alcian Blue. Bars = 20 μm

The cultured cells isolated from feline AT had a fibroblast-like morphology. The immune-phenotypes of the cells included high expression of cluster of differentiation (CD) 9 and CD44 and low expression of CD34 and CD45 (Fig. 1a). The fAT-MSCs had multilineage plasticity, as demonstrated by their potential for adipogenic, osteogenic, and chondrogenic differentiation. Adipogenic differentiation was evaluated by Oil Red O staining following 3 weeks of adipogenic induction. Matrix mineralization was evaluated by Alizarin Red S staining of fAT-MSCs following 3 weeks of osteogenic induction. Proteoglycans in cells were revealed by Alcian Blue staining after 3 weeks of chondrogenic induction (Fig. 1b).

### Clinical and mucosal healing of DSS-induced colitis

**Fig. 2:**
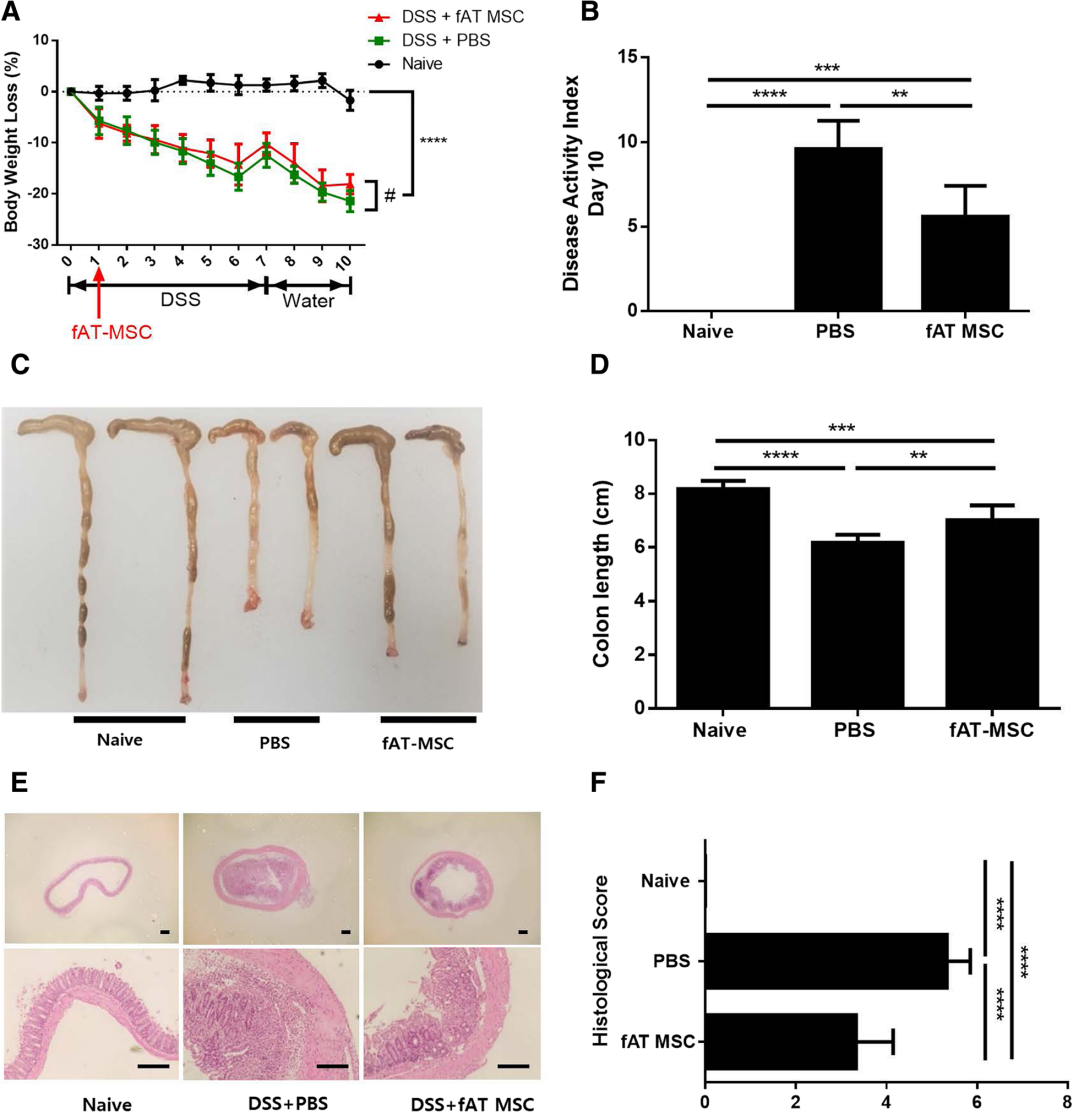
Intraperitoneally injected fAT-MSCs ameliorate IBD. 3% DSS water was administered to mice for seven days to induce colitis. fAT-MSCS were injected intraperitoneally one day after the administration of DSS. (*n* = 6 naïve, *n* = 8 PBS, n = 8 fAT-MSC) (**a**) Body weight, measured every day, was expressed as a relative change with respect to day 0 (**b**) DAI, and (**c, d**) Colon length were assessed, (**e, f**) H&E staining of the colon section and histological score are shown. Bars =100 μm. Results were shown as mean ± standard deviation (*P < 0.05, **P < 0.01, ***P < 0.001, ****P < 0.0001 by one-way ANOVA analysis and ^#^P < 0.05 by unpaired t test)

In this study, we first investigated whether fAT-MSCs exerted an anti-inflammatory effect on mice with DSS-induced colitis. On day 10, mice treated with DSS developed a severe acute illness, characterized by mild to moderate diarrhea, rectal bleeding, and depressed activity, accompanied by continuous weight loss (Fig. 2a, and b), and microscopic examination of the colon of the PBS group showed striking hyperemia, inflammation, necrosis, and shortening (Fig. 2c, and d) as well as histological changes with increased wall thickness, localized inflammatory cell infiltration, and epithelial ulceration (Fig. 2e, and f). However, the mice in the fAT-MSC-injected group showed amelioration of colitis compared with those in the PBS group (Fig. 2a, b, c, d, e, and f). After checking the degree of reduction in body weight for 10 days, there was no significant difference in weight loss between the PBS group and the fAT-MSC group from day 1 to day 9. However, on day 10, mice treated with fAT-MSCs showed a lower weight loss (*P* = 0.0322 by t -test comparison) than those treated with PBS (Fig. 2a). In addition, fAT-MSC-treated mice had lower clinical disease score (Fig. 2b). Moreover, on day 10, an autopsy was performed for histological evaluation of the colon. The results showed that fAT-MSC-treated mice had longer colon length (Fig. 2c, and d) than PBS-treated mice and showed significantly ameliorated colonic transmural inflammation, decreased wall thickness, reduced mucosal ulceration, and focal loss of crypts, all of which were associated with decreased disease scores and histological scores (Fig. 2e, and f).

### Effects of fAT-MSCs on immune responses in the colons of IBD model mice

**Fig. 3:**
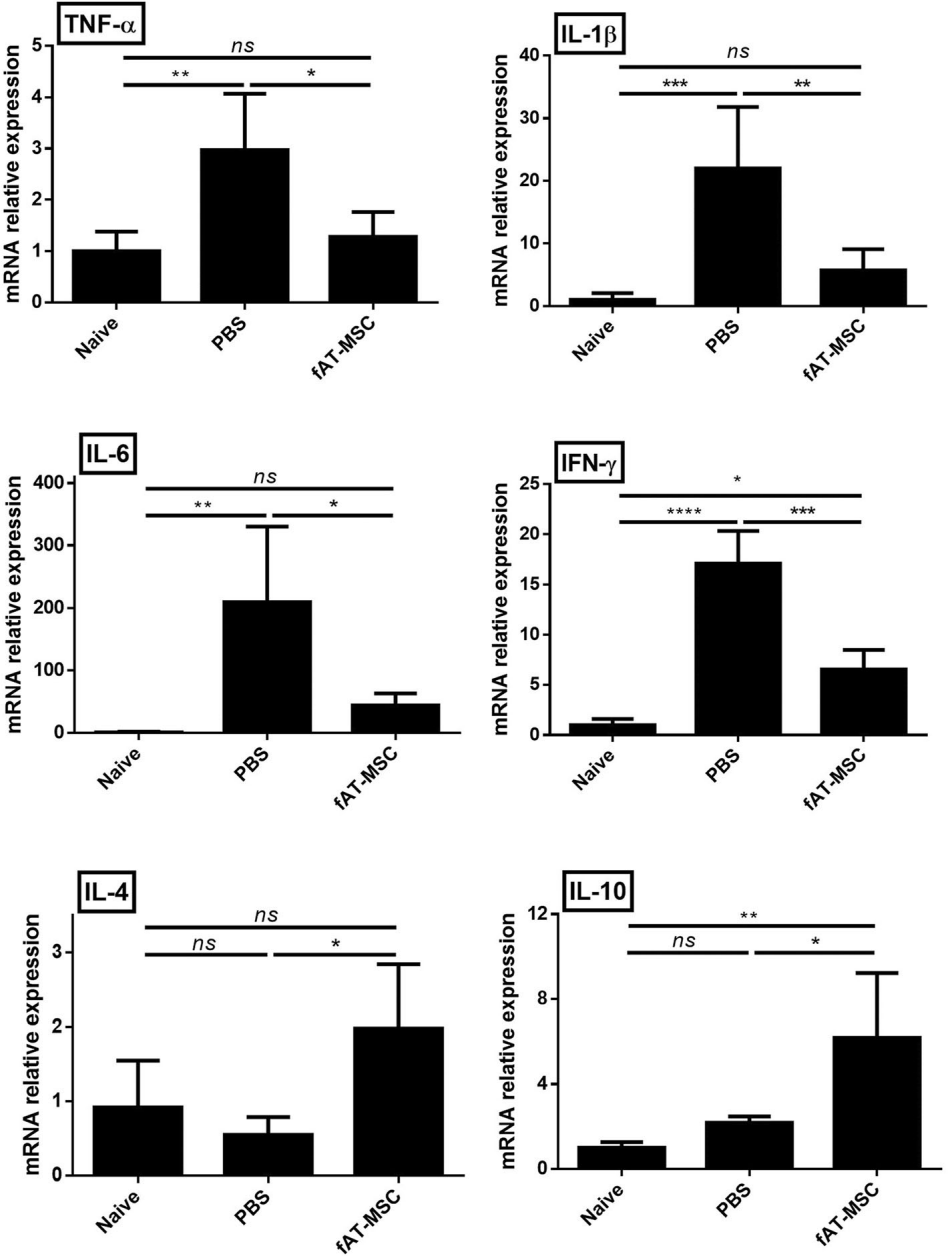
The fAT-MSCs inhibit inflammatory response in the colon. mRNA expression levels of pro- and anti-inflammatory cytokines in colon were determined by qRT-PCR (*n* = 6 naïve, *n* = 8 PBS, *n* = 8 fAT-MSC). Results were shown as mean ± standard deviation (*P < 0.05, **P < 0.01, ***P < 0.001 by one-way ANOVA analysis)

Because pro-inflammatory cytokines play important roles in the development of DSS-induced colitis, a possible mechanism of fAT-MSC therapy is suppression of the production of these cytokines in the colon. Therefore, we next investigated the effects of fAT-MSCs on the mRNA expression of inflammatory cytokines that are mechanistically linked to colitis in the colon of the same mouse as in the above experiments. The levels of tumor necrosis factor (TNF)-α, interleukin (IL)-1β, interferon (IFN)-γ, and IL-6 were markedly increased after DSS induction. However, the levels of these cytokines in colon tissues from DSS-induced mice that had been infused with fAT-MSCs were significantly lower than those in mice of the PBS group. The results indicated that the infusion of fAT-MSCs had inhibitory effects on the expression of TNF-α, IL-1β, IFN-γ, and IL-6, which are classically associated with DSS-induced colitis, in the colonic tissue. Conversely, the expression of the anti-inflammatory cytokines IL-4 and IL-10 increased in the fAT-MSC group relative to that in the PBS group (Fig. 3).

### Concentration of PGE_2_, secreted by fAT-MSCs, with and without NS-398

**Fig. 4:**
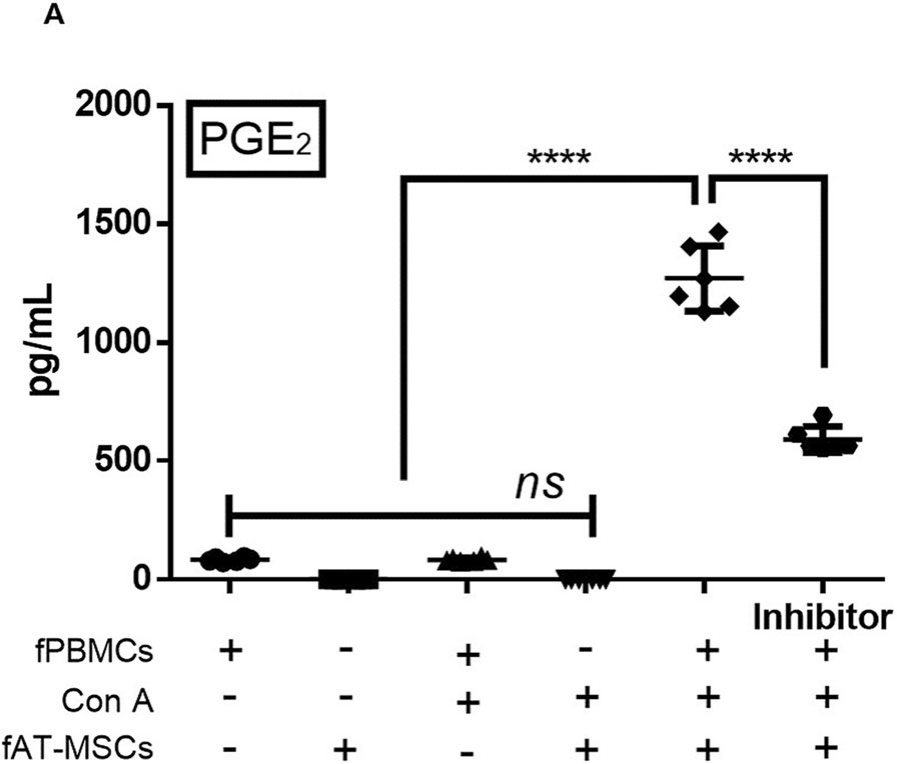
PGE_2_ concentration found in conditioned media from 48 h fAT-MSCs or fPBMCs only cultures and Con A-stimulated fPBMCs or fAT-MSCs only cultures and fAT-MSCs cocultured with Con A-stimulated feline PBMCs with and without NS-398, all measured by ELISA following manufacture’s protocol (*n* = 6 in each group). Inhibitor = NS-398, COX-2 inhibitor. Results were shown as mean ± standard deviation (****P < 0.0001 by one-way ANOVA analysis)

Previous studies have shown that PGE_2_ secreted from stem cells plays an important role in immune regulation [21, 22]. However, studies on PGE_2_ secreted by fAT-MSCs are insufficient. Therefore, to further assess the mechanisms underlying the fAT-MSC-dependent downregulation of pro-inflammatory cytokines and upregulation of anti-inflammatory cytokines in inflamed colon tissue, we established a fAT-MSC/feline peripheral blood mononuclear cell (fPBMC) co-culture protocol in vitro. Our findings confirmed that the concentration of PGE_2_ was increased in the supernatants of the fAT-MSC group cultured with concanavalin A (Con A)-stimulated feline PBMCs but was decreased in the group treated with NS-398, an inhibitor of the PGE_2_ synthesis-related enzyme, cyclooxygenase (COX)-2 (Fig. 4). To determine whether increased PGE_2_ was secreted from fAT-MSCs in co-cultured medium, the relative mRNA expression levels of *COX-2* in fAT-MSCs and fPBMCs were confirmed by qRT-PCR and agarose gel electrophoresis. The results showed that COX-2 was highly expressed in fAT-MSCs co-cultured with Con A-induced fPBMCs (Additional file 1). This suggested that PGE_2_ was secreted from fAT-MSCs rather than fPBMCs.

### Effects of fAT-MSCs on inflammatory responses in vitro

**Fig. 5:**
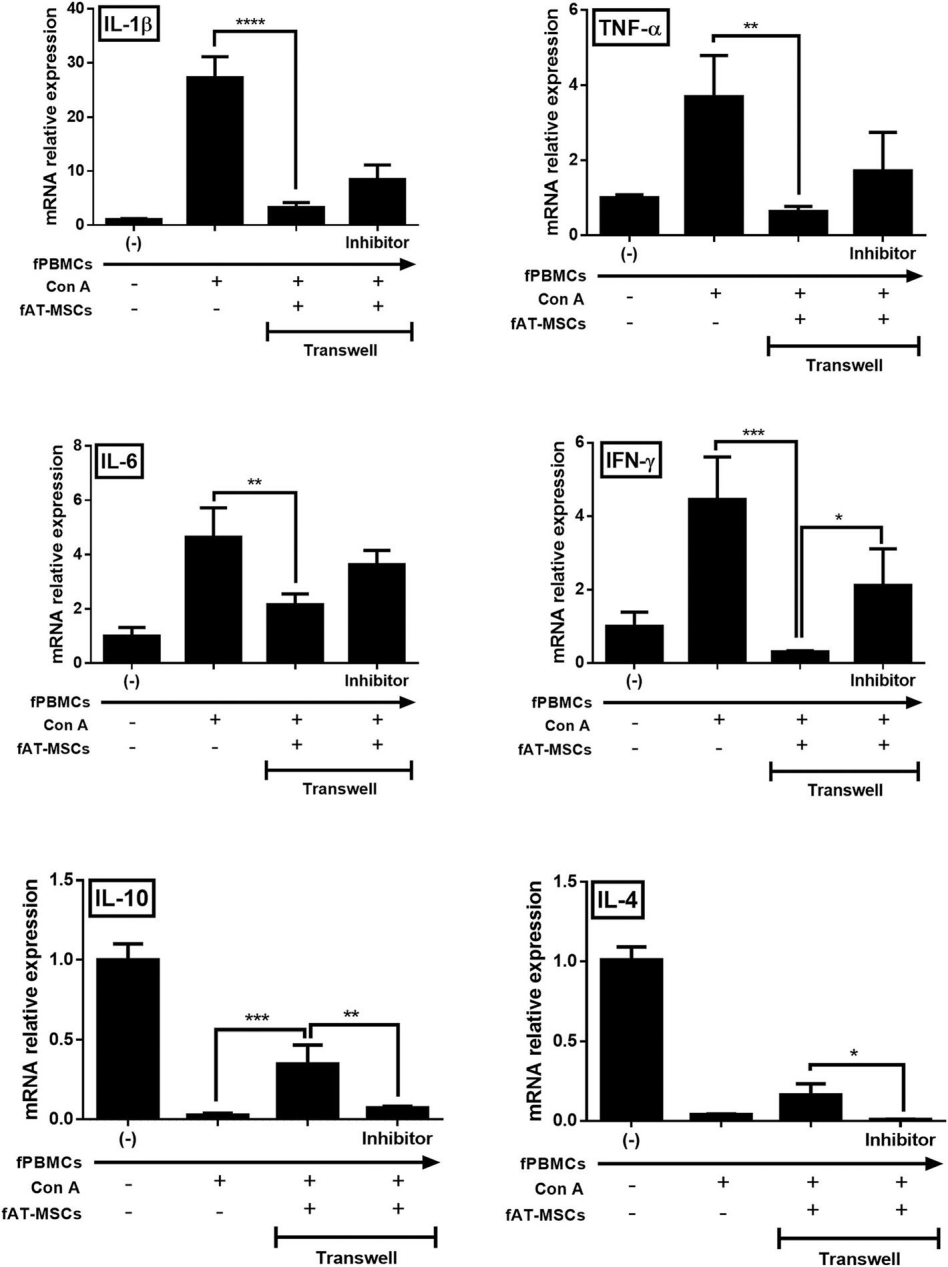
The fAT-MSCs inhibit inflammatory response in feline PBMCs. mRNA expression levels of pro-and anti-inflammatory cytokines in Con A-induced PBMCs were determined by qRT-PCR. PGE_2_ secreted from fAT-MSCs affects the degree of inflammatory cytokine mRNA level in feline PBMCs (n = 6 in each group). Inhibitor = NS-398, COX-2 inhibitor. Results were shown as mean ± standard deviation (*P < 0.05, **P < 0.01, ***P < 0.001, ****P < 0.0001 by one-way ANOVA analysis)

We then evaluated the anti-inflammatory effects of fAT-MSCs; pro- and anti-inflammatory cytokines were measured at the mRNA level in Con A-stimulated feline PBMCs. The expression of the pro-inflammatory cytokines, TNF-α, IL-1β, IFN-γ, and IL-6, decreased when Con A-stimulated feline PBMCs were co-cultured with fAT-MSCs. In contrast, the expression of anti-inflammatory cytokines, i.e., IL-4 and IL-10, increased. Next, we examined how the decreased secretion of PGE_2_ affected the cytokine-modulating effect of fAT-MSCs. Notably, both the pro- and anti-inflammatory cytokine-modulating effects of fAT-MSC were reduced in the NS-398 treatment group (Fig. 5).

### mRNA expression level of Forkhead box P3 (FOXP3) in fPBMCs co-cultured with fAT-MSCs

**Fig. 6:**
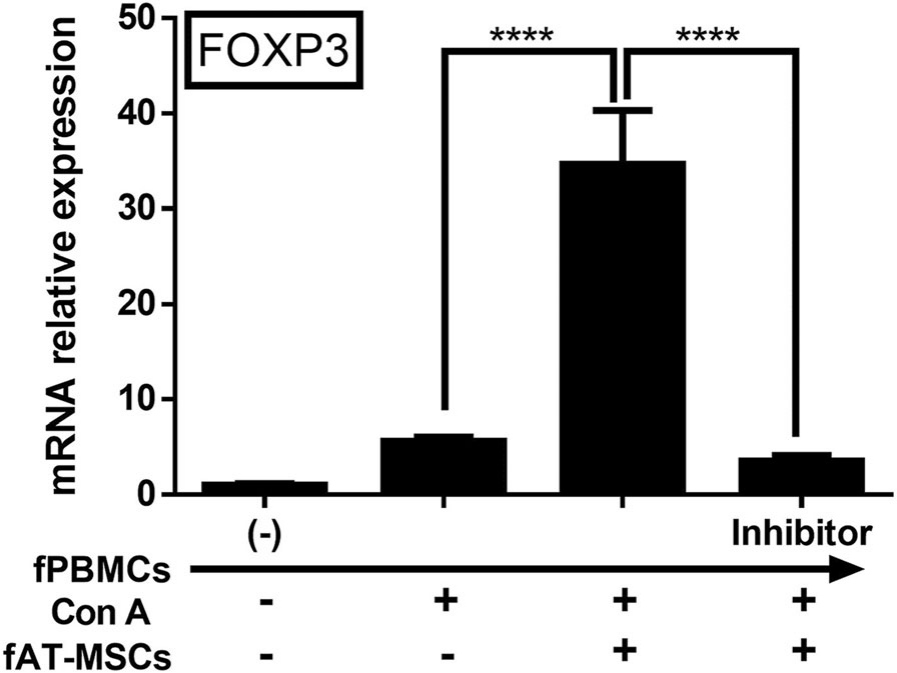
Change of mRNA expression of *FOXP*3 in feline PBMCs. PGE_2_ secreted from fAT-MSCs affects the degree of FOXP mRNA level in feline PBMCs (n = 6 in each group). Inhibitor = NS-398, COX-2 inhibitor. Results were shown as mean ± standard deviation (****P < 0.0001 by one-way ANOVA analysis)

PGE_2_ is known to be related to changes in T-cell polarization, and regulatory T cells (Tregs) are known to play important roles in alleviation of colitis [23–25]. Therefore, we determined the changes in T-cell phenotypes in the presence of different PGE_2_ concentrations. Because *FOXP3* is specifically expressed in naturally occurring Tregs, the extent of changes in *FOXP3* mRNA expression was confirmed by measuring changes in PGE_2_ concentrations. The expression of *FOXP3* mRNA increased with increasing PGE_2_ and decreased following treatment with NS-398 (Fig. 6).

### T-cell regulation by fAT-MSCs

**Fig. 7:**
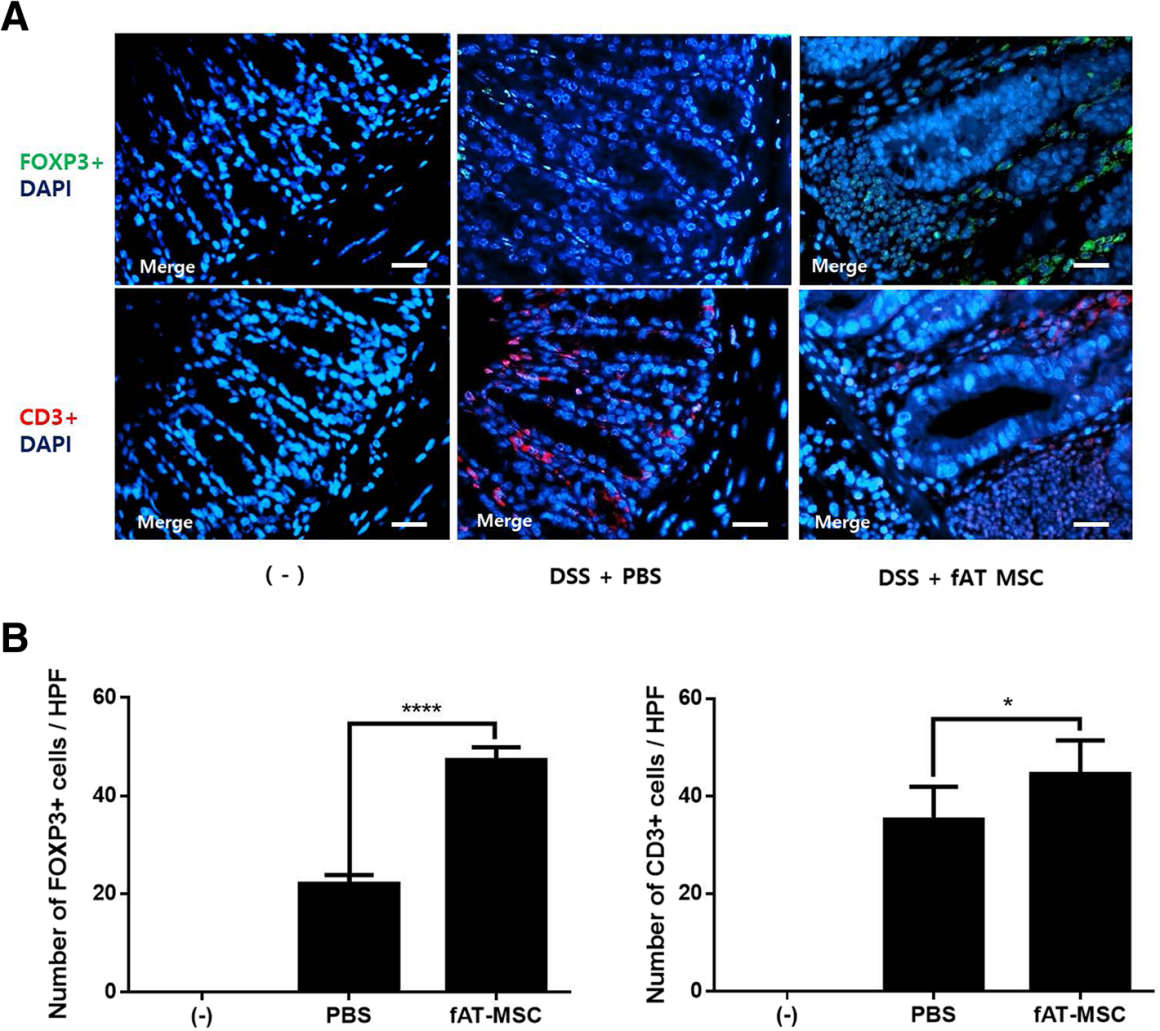
T-cell regulation by fAT-MSCs. Feline adipose tissue derived mesenchymal stem cells (fAT-MSCs) increase Tregs proportion in the inflamed colon (n = 6 naïve, n = 8 PBS, n = 8 fAT-MSC). (**a**) FOXP3 + (Green) and CD3 + (Red) cells detected in colon tissue section by immunofluorescence. Bar = 50 μm (**b**) The number of FOXP3+ and CD3+ cells in colon tissue. Results were shown as mean ± standard deviation. (*P < 0.05, ****P < 0.0001 by one-way ANOVA analysis)

Immunostaining was performed in the inflamed colon to examine whether the ratio of Tregs was also increased in vivo. CD3 and FOXP3 were stained separately but compared at the same sites in the same colon samples. Quantitative analysis of FOXP3+ and CD3+ cells, detected in colon tissue sections, showed that the extent of the increase was larger in FOXP3+ cells than in CD3+ cells in the fAT-MSC group compared with that in the PBS group (Fig. 7).

## Discussion

In this study, we aimed to determine whether administration of fAT-MSCs alleviated intestinal inflammation and whether regulation of inflammatory cytokines associated with colitis occurred through immune cell regulation via secretory factors from fAT-MSCs. Feline stem cells are immune privileged, partly due to the low expression of the major histocompatibility complex class II molecule [26–28]. Therefore, we performed in vivo experiments using immunocompetent mice to confirm that fAT-MSCs had anti-inflammatory effects through immune system control.

We showed that intraperitoneal administration of fAT-MSCs in mice with DSS-induced colitis alleviated the disease symptoms such as decreased activity, rectal bleeding, and stool consistency. In addition, the body weights of the mice, measured for 10 days, were not significantly different between the PBS and fAT-MSC groups from day 1 to day 9, but on day 10, the weight reduction in the fAT-MSC group was less than that in the PBS group. Although there was little difference in the weight between the PBS group and the MSC group, the clinical symptoms, colon length, and histological examination showed that the fAT-MSCs alleviated DSS-induced colitis.

Recent studies have shown that the expression of inflammatory mediators, such as cytokines, is an important factor in the progression of colitis [29]. In this experiment, we demonstrated that injection of fAT-MSCs reduced the expression of pro-inflammatory cytokines, such as TNF-α, IL-1β, IFN-γ, and IL-6. However, in the fAT-MSCs group, the levels of anti-inflammatory cytokines, such as IL-4 and IL-10, were upregulated in the injured colon. These results indicated a distinct correlation between the immunomodulatory potential of fAT-MSCs and their ability to ameliorate the inflammatory response in IBD.

In previous studies, MSCs were found to secrete certain cytokines, such as IDO, TGF-β, NO, and PGE_2_, among which, PGE_2_ has been shown to be pivotal for the anti-inflammatory effect of MSCs in several inflammatory disease models, including wound disease, brain disease, arthritis, lung injury, periodontitis, and colitis [30–36]. Such anti-inflammatory effects are mediated through immune regulation; in the intestinal tract, immunomodulation occurs mainly via T cells [37–40]. Therefore, in this study, we hypothesized that PGE_2_ secreted from fAT-MSCs plays an important role in immune regulation and that reduction of PGE_2_ secretion from stem cells decreases the immunoregulatory capacity of fAT-MSCs. For this experiment, NS-398 (a selective COX-2 inhibitor; which has not been used in fAT-MSCs but has been used in various cells) [41–43] was used as an inhibitor of PGE_2_ secretion by fAT-MSCs. To prove this, we directly monitored protein concentration of PGE_2_ secreted from fAT-MSCs in conditioned medium following co-culture of fPBMCs and fAT-MSCs. The result confirmed that the concentration of PGE_2_ was high in the medium of Con A stimulated-PBMCs co-cultured with MSCs, and conversely, in the group treated with NS-398, the PGE_2_ concentration was decreased. In addition, fAT-MSCs co-cultured with fPBMCs stimulated with Con A (a T-cell mitogen activator) showed reduced expression of pro-inflammatory cytokines, such as TNF-α, IL-1β, IL-6 and IFN-γ. However, in the NS-398-treated group, the overall anti-inflammatory effects of fAT-MSCs were decreased, and especially, mRNA expression of IFN-γ was significantly increased in the inhibitor group than in the fAT-MSCs group. In the case of IL-10 and IL-4, known to be anti-inflammatory or immunosuppressive cytokines, Con A -induced fPBMCs showed an increasing tendency when cultured with fAT-MSC. However, the NS-398-treated group showed the opposite tendency. Taken together, fAT-MSCs have cytokine-modulating effects on immune cells, and PGE_2_ indirectly plays a major role in this regulatory effect.

Diverse regulatory mechanisms cooperate to maintain intestinal homeostasis [44, 45], and disruption of these pathways may lead to inappropriate immune responses to intestinal communities [46, 47], thereby contributing to pathogenesis. Several studies have shown that bowel homeostasis is closely related to Treg activation [48–50]. Moreover, colonic Tregs recognize and suppress immune responses against antigens, including commensal bacteria and food [51, 52]. In particular, FOXP3+ Tregs, most of which are CD4+ T cells, are potent mediators of dominant self-tolerance in the periphery and can suppress the activation, proliferation, and effector functions of a wide range of immune cells, including natural killer cells, B cells, antigen-presenting cells, and T cells [53]. In addition, in in vivo and in vitro studies, IL-10 was significantly increased in the fAT-MSCs group when anti-inflammatory cytokines were measured, and many studies have reported that IL-10 is closely related to regulatory T cells [54, 55]. Therefore, understanding the mechanisms responsible for the development of *FOXP3+* Tregs in the intestine of patients with IBD could provide new therapeutic options.

The mRNA expression levels of *FOXP3*, a Treg lineage-specification factor [56, 57], were further confirmed in vitro; *FOXP3* expression increased in the fAT-MSC group but decreased in the COX-2 inhibitor group. In addition, in vivo, fAT-MSCs blocked the infiltration of CD3+ T cells and increased the FOXP3+ Treg population in the injured colons of DSS-treated mice. These results suggested that the increased number of colonic Tregs in the fAT-MSC-treated group was associated with PGE_2_ secreted from fAT-MSCs.

Although we could not rule out the possibility of the contribution of other factors secreted from fAT-MSCs to the FOXP3+ Treg proliferation in mice with colitis, our findings collectively suggested that fAT-MSCs inhibited inflammation by regulatory T cells via a paracrine mechanism and that PGE_2_ secreted by fAT-MSCs may play an important role in increasing Tregs in mice with DSS-induced colitis.

## Conclusions

PGE_2_ released by fAT-MSCs alleviated DSS-induced colitis in mice by inducing an increase in the Treg population. Our data indicated that regulation of PGE_2_ production modulated Treg development and function, thereby suggesting attractive therapeutic strategies, such as targeting PGE_2_-activated Tregs in the treatment of IBD. Taken together, our findings suggested that fAT-MSCs may be potential candidates for cell-based clinical therapy in cats with IBD.

## Methods

### Cell preparation and characterization

With the consent provided written of the owner, Adipose tissue was obtained from a healthy, adult, female, domestic short-haired cat (1-year-old, 5.5 kg) during ovariohysterectomy at Seoul National University Veterinary Medicine Teaching Hospital; MSCs were isolated as previously described [58]. Briefly, the tissue sample was washed four times in Dulbecco’s PBS (PAN-Biotech, Aidenbach, Germany) with 1% penicillin-streptomycin (PS; PAN-Biotech), cut into small pieces, and digested for 1 h at 37 °C with collagenase type 1A (1 mg/mL; Sigma-Aldrich, St. Louis, MO, USA). The enzymatic activity was inhibited by Dulbecco’s modified Eagle’s medium (DMEM; PAN-Biotech) containing 20% fetal bovine serum (FBS; PAN-Biotech). Following centrifugation at 1200×*g* for 5 min, the pellet was filtered through a 70-μm Falcon cell strainer (Fisher Scientific, Pittsburgh, PA, USA) to remove debris; erythrocytes in the pellet were eliminated by adding 1 mL red blood cell (RBC) lysis buffer (Sigma-Aldrich), and the cell solution was incubated for 5 min at 25 °C. Pellets were resuspended in DMEM containing 20% FBS and 1% PS and transferred to 100-mm dishes at a density of 3000 cells/cm^2^. Transferred cells were incubated in DMEM containing 20% FBS at 37 °C in a humidified atmosphere of 5% CO_2_, and the medium was replaced every 2–3 days until the adhered cells showed a fibroblast-like morphology and reached 70–80% confluence. Thereafter, the cells were repeatedly subcultured under standard conditions. Cells were characterized by flow cytometry using antibodies against the following proteins: CD9, CD44 (GeneTex, CA, USA), CD34-phycoerythrin (PE), and CD45-fluorescein isothiocyanate (FITC; eBiosciences, San Diego, CA, USA). For CD9 and CD44, indirect immunofluorescence was performed using goat anti-mouse IgG-FITC and goat anti-rat IgG-PE (Santa Cruz Biotechnology, Santa Cruz, CA, USA), respectively [43, 59]. Characterization was conducted using FlowJo 7.6.5 software (TreeStar, Inc., Ashland, OR, USA). Cellular differentiation was evaluated using special kits (StemPro Adipogenesis Differentiation, StemPro Osteogenesis Differentiation, and StemPro Chondrogenesis Differentiation kits; Gibco/Life Technologies, Mulgrave, Australia) according to the manufacturer’s instructions, followed by Oil Red O staining, Alizarin red staining, and Alcian blue staining.

### Animal experiments and cell transplantation

Male C57BL/6 mice, aged 6–8 weeks, were purchased from Nara Biotech (Seoul, Korea). All experimental procedures involving animals were approved by the Institutional Animal Care and Use Committee of SNU (protocol no. SNU-171121-5), and all protocols were in accordance with approved guidelines. Environmental conditions were maintained at a constant temperature of 25 °C and humidity of 50% with a 12-h light/dark cycle. For environmental enrichment, 3–4 mice were raised in polycarbonate cages (W324 × D221.5 × H130 mm) containing clean bedding (shavings; Nara Biotech), cardboard boxes, and tunnels. All the mice were supplied with sterilized maintenance mouse food and fresh water ad libitum. The studies were conducted using 22 animals, and mice were randomly divided into three groups, each containing 6–8 mice (*n* = 6 naïve, *n* = 8 PBS, n = 8 fAT-MSC). At the start of the experiments, the health status of the mice was evaluated by weight, vitality, and defecation, and the experiments were carried out with mice with no abnormal symptoms. Colitis was induced by administration of 3% DSS (36–50 kDa; MP Biomedical, Solon, OH, USA) in drinking water ad libitum from day 0 to day 7. On day 1, the following procedure was performed: fAT-MSCs (2 × 10^6^ cells in 200 μL PBS) or an equivalent PBS volume was injected intraperitoneally into the mice. During housing, animals were monitored once daily for health status. The mice were sacrificed on day 10, and colon tissues were collected for subsequent processing. On day 10 of the study, all the mice were humanely euthanized with injection of xylazine and inhalation of CO_2_. A completed ARRIVE guidelines checklist is included in Checklist S1.

### Assessment of the severity of colitis

The severity of colitis was assessed by scoring the clinical disease activity, including body weight loss, stool consistency, rectal bleeding, and general activity (Table 1). The combined DAI ranged from 0 to 16.

**Table 1:** Disease Activity Index

Parameters	Changes	Scores
Body weight loss	None	0
	< 10%	1
	10–15%	2
	15–20%	3
	> 20%	4
Stool consistency	None	0
	Mild diarrhea	2
	Moderate to severe diarrhea	4
Rectal bleeding	None	0
	Mild bleeding	2
	Moderate to severe bleeding	4
General activity	Normal	0
	Mildly depressed	2
	Moderately to severely depressed	4

### Histological analysis

Colon segments were fixed in 10% formaldehyde for 48 h, and paraffin-embedded sections were prepared for hematoxylin and eosin (H&E) staining. Histological sections of distal colon were scored blindly by an independent researcher. And Histological scores were assessed as means ± standard deviations of the different groups of colon segments. The severity of symptoms was calculated by scoring tissue inflammation and tissue damage grade (Table 2). The combined histological score for severity of colitis ranged from 0 to 6.

**Table 2:** Histological colitis severity

Parameters	Changes	Scores
Tissue inflammation	none	0
	inflammatory cells in the lamina propria	1
	inflammatory cells extending into the submucosa	2
	inflammatory cells infiltrate Transmural extension	3
Tissue damage	None	0
	Discrete lymphoepithelial lesions	1
	Mucosal erosions	2
	Discrete deeper structures of the bowel wall	3

### Co-culture of feline PBMCs with fAT-MSCs

Blood samples were obtained from the jugular vein of two healthy adult cats with the consent provide written of the owners, and blood (5 mL each) was collected into sterile CPDA tubes. Feline blood was diluted with an equal volume of PBS and layered over Ficoll-Paque PLUS (GE Healthcare Life Sciences, Piscataway, NJ, USA) in a conical tube. After centrifugation at 780×*g* for 30 min, the buffy coat layer was carefully collected. The collected sample was resuspended with RBC lysis buffer and incubated at 25 °C for 5 min. After adding PBS, samples were centrifuged at 850×*g* for 10 min, washed, and centrifuged again; The fPBMCs were plated at a density of 1 × 10^6^ cells/well in 6-well plates (SPL Life Science, Pocheon, Korea), resuspended in DMEM containing 20% FBS and 1% PS, and stimulated with 5 μg/mL Con A (Sigma-Aldrich) for 6 h before further experiments [43]. Then, 2 × 10^5^ fAT-MSCs were seeded onto 0.4-μm pore-sized Transwell inserts (SPL Life Science). Additionally, the PGE_2_ inhibitor NS-398 (5 μM; Enzo Life Science) was added to the medium in the inhibitor group. The appropriate dose of NS-398 in these experiments was determined based on a previous study on the effects of NS-398 in feline cells [43]. After incubation for 48 h, total RNA and proteins were extracted from the PBMCs and fAT-MSCs following collection by scraping and 2 ml of the culture supernatant was collected for enzyme-linked immunosorbent assay (ELISA) for PGE_2_.

### RNA extraction, cDNA synthesis, and quantitative real-time reverse transcription polymerase chain reaction (qRT-PCR)

For in vivo experiments, six colon tissues were collected from each group, and for in vitro experiments, five replicates each were analyzed for fPBMCs and fAT-MSCs for each group. Total RNA was extracted from homogenized colon tissue, fPBMCs, or fAT-MSCs using an Easy-BLUE Total RNA Extraction kit (Intron Biotechnology, Seongnam, Korea) according to the manufacturer’s instructions. Extracted RNA was converted into cDNA using LaboPass M-MuLV Reverse Transcriptase (Cosmo Genetech, Seoul, Korea) following the manufacturer’s instructions. Samples were analyzed in duplicate using 10 μL AMPIGENE qRT-PCR Green Mix Hi-RO with SYBR Green dye (Enzo Life Science, Lausen, Switzerland), 7.4 μL PCR-grade dH_2_O, 0.8 μL forward and reverse primers (Bionics, Seoul, Korea; Table 3), and 1 μL template cDNA. Cytokine mRNA levels were quantified by comparison with that of glyceraldehyde 3-phosphate dehydrogenase.

**Table 3:** Sequences of PCR primers used in this study

Gene	Forward (5′-3′)	Reverse (5′-3′)	Reference
fGAPDH	ACGATGACATCAAGAAGGTG	CACACCAGGAAATGAGCTTG	[51]
f*FOXP*3	GCCTGCCACCTGGAATCAAC	GTGTGCTGGGGCTTGGGA	[52]
fIFN-γ	TACACAAGTTTTATTTTCGCTTTCC	TGCTACATCTGGATTACTTGCATTA	[51]
fIL-6	TGAAAAAGGAGATGTGTGACAACTA	CCTGAAGACCAGTAGTGATTCTTGT	[51]
fIL-10	CCTTTAGTAAGCTCCAAGAGAAAGG	CAGATTTTCATCTTCATTGTCATGT	[51]
fTNF-α	GACACTCAGATCATCTTCTCGAACT	GACCTGGGAGTAGATGAGGTACAG	[51]
fIL-1β	CATACAGTCACAGGACTACACGTTC	TTGATGCACAACACTACTGGTATCT	This study
fIL-4	GGCAGATCTATACACATCACAACTG	GCTTTGAGTATTTCTTTTGCATGAT	This study
mGAPDH	TCATTGACCTCAACTACAA	ACACCAGTAGACTCCACGT	[51]
mINF-γ	CACAGTCATTGAAAGCCTAGAAAGT	AGTTCCTCCAGATATCCAAGAAGAG	This study
mIL-6	CGCACTAGGTTTGCCGAGTA	CCTTTCTACCCCAATTTCCA	This study
mIL-10	GTGATTTTAATAAGCTCCAAGACC	GATCATCATGTATGCTTCTATGCAG	This study
mTNF-α	CCCTCACACTCAGATCATCTTCT	GCTACGACGTGGGCTACAG	[51]
mIL-1β	GTCTTTCCCGTGGACCTTC	TGTTCATCTCGGAGCCTGT	[51]
mIL-4	TAGTTGTCATCCTGCTCTTCTTTCT	CGATGATCTCTCTCAAGTGATTTTT	This study
fCOX-2	CGATTCAGTCTCTCATCTGCAATAA	TCAGTTGAACGTTCTTTTAGCAGTA	[51]

### Determination of PGE_2_ expression by fAT-MSCs in the conditioned medium

Supernatants from fPBMCs and fAT-MSCs culture medium were obtained after 48 h of incubation and used for protein analysis. PGE_2_ secreted from fAT-MSCs in the conditioned medium was quantified using an ELISA kit (Enzo Life Science) according to the manufacturer’s instructions.

### Immunofluorescence analysis

Paraffin-embedded colon tissue sections were cut into 4-μm-thick sections. Sections were deparaffinized in xylene and rehydrated sequentially in 100, 95, and 80% ethanol solutions; antigen retrieval was carried out using 10 mM citrate buffer (Sigma-Aldrich). After washing, the sections were blocked with a blocking buffer containing 1% bovine serum albumin in PBST for 30 min. The sections were incubated overnight at 4 °C with antibodies against FOXP3 (1:50; Santa Cruz Biotechnology) or CD3 (1:50; Santa Cruz Biotechnology). After three washes, the slides were incubated with secondary antibody. The colon sections, stained with an antibody against either FOXP3 or CD3, were washed three times and incubated with fluorescein-conjugated secondary antibodies (1:200; Santa Cruz Biotechnology) or Texas red-conjugated secondary antibodies (1:200; Santa Cruz Biotechnology) for 1 h at room temperature in the dark. Colon sections, stained with antibodies against either FOXP3 or CD3, were washed three times and mounted in VECTASHIELD mounting medium containing 4′,6-diamidino-2-phenylindole (DAPI; Vector Laboratories, Burlingame, CA). The samples were observed using an EVOS FL microscope (Life Technologies, Darmstadt, Germany), and the immuno-reacted cells were counted in 20 random fields per group.

### Statistical analysis

Data are shown as the mean ± standard deviation. Mean values among different groups were compared by one-way analysis of variance using the GraphPad Prism 6.01 (GraphPad, Inc., La Jolla, CA). A *P*-value < 0.05 was considered statistically significant.

## Additional file

Additional file 1: Relative mRNA expression of COX-2 in feline PBMCs and fAT-MSCs. (A) mRNA level of PGE2 were measured in feline PBMCs (Black bars) and fAT-MSCs (Gray bars). This data shows that mRNA levels of COX-2 are highly expressed in fAT-MSCs cocultured with Con A-stimulated PBMCs (*n* = 6 in each group). Results are shown as mean ± standard deviation (****P < 0.0001 by one-way ANOVA analysis) (B) PCR amplification of COX-2 in fAT-MSC and feline PBMCs in cocultured group. fAT-MSCs; Lane 1, 2 and 3, fPBMCs; Lane 4, 5 and 6. All experiments were conducted in triplicate independently. (PDF 57 kb)

## Abbreviation

APC: Antigen Presenting Cell
CD: Cluster of Differentiation
DAPI: 4′,6-diamidino-2-phenylindole
fAT-MSC: Feline Adipose Tissue-Derived Mesenchymal Stem Cells
FITC: Fluorescein Isothiocyanate
FOXP3: Forkhead box P3
fPBMC: Feline Peripheral Blood Mononuclear Cell
GAPDH: Glyceraldehyde 3-Phosphate Dehydrogenase
H&E: Hematoxylin and Eosin
IBD: Inflammatory Bowel Disease
IDO: Indoleamine-pyrrole 2,3-Dioxygenase
IFN: Interferon
IL: Interleukin
MSC: Mesenchymal stem cell.
NK: Natural Killer
NO: Nitric Oxide
PBMC: Peripheral Blood Mononuclear Cell
PE: Phyco-Erythrin
PGE: Prostaglandin E
RBC: Red Blood Cell
TGF-β: Transforming Growth Factor-β
TNF-α: Tumor Necrosis Factor-alpha
Treg: Regulatory T cell
