# Supplementary figures and images for: Prostaglandin E2 secreted from feline adipose tissue-derived mesenchymal stem cells alleviate DSS-induced colitis by increasing regulatory T cells in mice
Source: BMC Vet Res. 2018 Nov 20;14:354. doi: 10.1186/s12917-018-1684-9 (PMC6245895; doi:10.1186/s12917-018-1684-9)

A

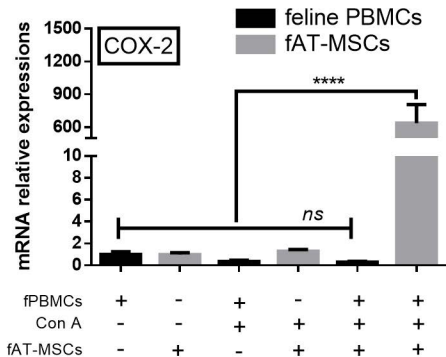

B

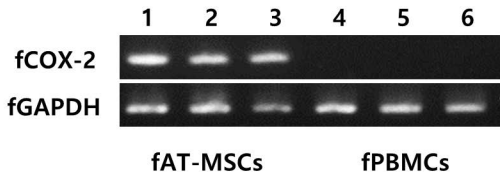

Supplement: Supplementary file 1 — Relative mRNA expression of COX-2 in feline PBMCs and fAT-MSCs. (A) mRNA level of PGE2 were measured in feline PBMCs (Black bars) and fAT-MSCs (Gray bars). This data shows that mRNA levels of COX-2 are highly expressed in fAT-MSCs cocultured with Con A-stimulated PBMCs (n = 6 in each group). Results are shown as mean ± standard deviation (****P < 0.0001 by one-way ANOVA analysis) (B) PCR amplification of COX-2 in fAT-MSC and feline PBMCs in cocultured group. fAT-MSCs; Lane 1, 2 and 3, fPBMCs; Lane 4, 5 and 6. All experiments were conducted in triplicate independently. (PDF 57 kb) [file 12917_2018_1684_MOESM1_ESM.pdf]
